# Postoperative thrombocytopenia and subsequent consequences in acute type A aortic dissection

**DOI:** 10.1080/07853890.2023.2281653

**Published:** 2023-12-10

**Authors:** Zhiwei Tang, Yongfeng Shao

**Affiliations:** Department of Cardiovascular Surgery, The First Affiliated Hospital with Nanjing Medical University, Nanjing, China

**Keywords:** Postoperative thrombocytopenia, type a acute aortic dissection, mortality and morbidity

## Abstract

**Objectives:**

To ascertain if postoperative thrombocytopenia following open aortic surgery with a median sternotomy can predict early- and intermediate-term morbidity and mortality.

**Methods:**

From January 2018 to December 2022, a comparison was made between patients who had and didn’t have postoperative thrombocytopenia (defined as a nadir < 75 × 103/μL after 72 h of open aortic surgery with median sternotomy). Intermediate-term mortality during follow-up was the main result, with cerebrovascular accident and acute renal injury requiring dialysis as secondary events. Inverse probability treatment weighting (IPTW) was used to account for selection bias between groups. The Kaplan-Meier method with the log-rank test was used to assess intermediate-term survivals following IPTW modification. To identify the nonlinear link between platelet nadir and mortality probability, a generalized additive mix model was applied. To help increase power in testing for the overall effect of platelet nadir on outcomes in the generalized additive mix model, the hazard ratios and 95% CIs for each subgroup and their interactions were examined.

**Results:**

The study included 457 patients, 347 male (75.9%), with mean age of 54 ± 12 years. The last follow-up was done on April 14^th^, 2023 and the median follow-up time was 16 (6-31) months. Following IPTW, patient characteristics were balanced among cohorts. Platelet nadir was found to be significantly inversely related to early-term mortality (IPTW-adjusted hazard ratio = 0.968 (0.960, 0.977), *p* < 0.001), and AKI requiring dialysis (IPTW-adjusted hazard ratio = 0.979 (0.971, 0.986), *p* < 0.001). A nonlinear relationship between platelet nadir and mortality risk probability during follow-up visually showed that the likelihood of mortality decreased with platelet nadir increased. In confounder-adjusted survival (‘postoperative thrombocytopenia not acquired’ vs ‘postoperative thrombocytopenia’; HR: 0.086 [95% CI: 0.045-0.163]; *p* < 0.01) analysis, non-acquired postoperative thrombocytopenia was associated with a lower risk of mortality, and the treatment benefit was validated in IPTW-adjusted analysis, which showed an HR of 0.067.

**Conclusions:**

Early postoperative thrombocytopenia following type A aortic dissection surgery is a risk factor for morbidity and mortality. Because postoperative thrombocytopenia can indicate a poor prognosis, monitoring early postoperative platelets helps identify individuals who may develop late postoperative problems, which is performed by this affordable biomarker.

## PLAIN LANGUAGE SUMMARY

What is the context?

1. The most common complications of acute type A aortic dissection included postoperative bleeding, acute kidney injury (AKI), rethoracotomy for hemostasis due to hemorrhage, stroke and even death.
2. It is unknown that platelets are associated with morbidity and mortality in type A aortic dissection.

What is new?

1. The present study suggests that early postoperative thrombocytopenia following type A aortic dissection surgery is a risk factor for short- and intermediate-term morbidity and mortality.
2. Furthermore, a nonlinear relationship between platelet nadir and mortality risk probability during follow-up visually showed that the likelihood of mortality decreased with platelet nadir increased.
3. Especially, in confounder-adjusted Kaplan-Meier survival analysis, postoperative thrombo­cytopenia was associated with a higher risk of mortality, and the effect was also validated in IPTW-adjusted analysis.

What is the impact?

This study provides further evidence that the platelet count represents a reliable early monitoring tool for the predictive value in the prognosis of acute type A aortic dissection.

## Introduction

Stanford acute type A aortic dissection (ATAAD) is a potentially fatal cardiovascular emergency in adults, with substantial morbidity and mortality [1]. The most common complications included postoperative bleeding, acute kidney injury (AKI), rethoracotomy for hemostasis due to hemorrhage, stroke and even death. Despite this, not enough attention has been made to identifying patients who were at high risk of poor outcomes.

Platelets are thought to fall below 100 × 10^9^/L during cardiac surgery requiring cardiopulmonary bypass (CPB). On the second and third postoperative days, platelet counts were found to be at their lowest [2,3]. In patients undergoing non-emergent cardiac surgery, postoperative thrombocytopenia is associated with increased morbidity and mortality, as well as a poor prognosis, with a 3-fold greater risk of postoperative acute renal injury and a 5-fold increased risk of postoperative mortality [4].

While platelets are crucial for coagulation, their vascular relevance has been attributed to their function in stroke and AKI [5]. The increased risk of first stroke was found in Chinese hypertensive individuals with low Platelet in a multicenter, randomized, controlled trial [6]. It’s plausible that different types of endothelium damage could encourage platelet adhesion and the release of platelet components [7]. Low PLT may therefore indicate endothelial damage and platelet adhesion.

Preoperative mean platelet volume to platelet count ratio has been demonstrated to predict clinical outcomes [8]. Similarly, our prior research has discovered that preoperative platelet count was a significant predictor of postoperative mortality and multiple organ dysfunction syndrome [9]. Furthermore, recent studies have mainly focused on the predictive efficacy of postoperative platelet counts on short-term postoperative mortality in type A acute aortic dissection [10,11]. However, the relationship between postoperative thrombocytopenia and the development of postoperative intermediate-term outcomes in ATAAD patients is uncertain. Hence, we wanted to see if early postoperative thrombocytopenia within 72 h of open surgery for aortic repair affected late postoperative mortality and morbidity in ATAAD patients.

## Materials and methods

### Patient population

Patients were eligible if they were older than 18 and diagnosed with TAAD and admitted to the emergency medicine center of our hospital. Patients were excluded if either patient refused operation or if patients had medications that may contribute to thrombocytopenia 7 days before surgery. The First Affiliated Hospital Ethical Committee of Nanjing Medical University gave its approval to the project. According to the Stanford classification criteria, computed tomographic angiography was primarily used to diagnose ATAAD when symptoms first appeared and fewer than 14 days had passed.

### Transfusion indications

Per institution protocol, indications for single donor platelet transfusion included life-threatening active bleeding and a platelet count≤ 50 × 10^9^/L. No patients receive platelet substitution therapy.

### Laboratory assessment

In accordance with institutional practice, platelets are routinely checked every day for the first three days following surgery, and then again if the patient’s platelets fell below critical levels. Perhaps the patient might experience active bleeding following surgery and require repeated platelet measurements on a single day. In either case, a predictive variable was chosen based on the nadir value of platelet readings.

### End points and definitions

The primary end point was mortality during follow-up. The definition of Intermediate-term mortality was that the date of mortality occurred more than 30 days after admission for emergency surgery. Additional prespecified end points included stroke, AKI requiring dialysis and mortality during hospitalization. Stroke was defined as an intense episode of a central or global neurologic dysfunction caused by vascular injury to the brain resulting from hemorrhage or infarction. Stroke was diagnosed by a CT or MR scan. The identification of covert (asymptomatic) brain infarction was not routinely neuroimaged. Just in patients in whom stroke was suspected, neuroimaging was done according to the standard of care at Intensive Care Unit or inpatient ward at the discretion of the treating physician. If a patient had an anomalous urine volume or blood creatinine levels after surgery and had AKI that required dialysis, we would consult a qualified nephrologist before deciding whether to perform dialysis. Postoperative thrombocytopenia was defined as a platelet nadir <75 x 10^3^/μL within 72 h postoperatively. This cut-off value was selected based on the value of the definition of postoperative thrombocytopenia reported in the previous literature [12]. However, when mortality or morbidity preceded the nadir platelet count within 72 h postoperatively, we used the lowest platelet values ahead of those as predictors in multivariate analyses.

### Candidate predictors

All candidate predictors that were associated with mortality and morbidity were selected based on detailed literature reviews and clinical evidence within the confines of available data. Age, gender, hypertension, and laboratory tests were included in baseline characteristics. Transfusion data included packed red cell transfusions, single donor platelet transfusions, fresh frozen plasma, and cryoprecipitate. Prognostic data included transient ECMO treatment, stroke, AKI requiring dialysis, and mortality.

### Statistical analysis

Given the observational nature of our dataset, inverse probability treatment weighting (IPTW) was performed to address confounding factors between two cohorts (Table 2). Covariates included in the IPTW model were age, history of stroke, smoke, diabetes mellitus, chronic obstructive pulmonary diseases, intubation before entering the operative room, body mass index, hypertension, shock, gender, and some laboratory tests. Design-based Kruskal–Wallis test was used to compare continuous data after IPW adjustment and Rao-Scott χ2 test was used to compare categorical data using the survey package for R. The survival curve was abridged when number at risk was fewer than 10. To model the link between results and platelet nadir, we use generalized additive models (GAMs), a flexible variant of generalized linear models (GLM). Kaplan-Meier curves were used to assess the impact of platelet nadir on postoperative survival in the beginning (log-rank test). The area under the receiver operating characteristic curve (AUROC) was used to measure discrimination performance of postoperative platelet nadir on intermediate-term mortality. A Hosmer-Lemeshow test was carried out to evaluate the model’s goodness of fit. Using Cox proportional hazards models and adjusting for relevant factors, the hazard ratios (HRs) and 95% confidence intervals (CIs) for the risk of mortality related with platelet nadir were calculated. To help increase power in testing for the overall effect of platelet nadir on outcomes in Cox hazard proportional models, the hazard ratios and 95% CIs for each subgroup and their interactions were examined. The models’ covariates were chosen based on published research and clinical experience. Statistical significance was set at *p* < 0.05 (two-sided). R version 4.0.3 was used to conduct all analyses (R Foundation for Statistical Computing, Vienna, Austria) (Table 1).

**Table 1.** Perioperative raw data of two groups.

	Group A*(*N* = 248)	Group B (*N* = 209)^†^	*P* value
Age (year), median (IQR)	56 (48, 64.25)	51 (43, 60)	<0.001
History of stroke, n (%)	15 (6.0)	8 (3.8)	0.386
Smoke, n (%)	56 (22.6)	44 (21.1)	0.779
DM, n (%)	7 (2.8)	5 (2.4)	1.000
COPD, n (%)	6 (2.4)	3 (1.4)	0.677
Intubation, n (%)	6 (2.4)	1 (0.5)	0.193
BMI, median (IQR)	25.03 (22.49, 27.68)	25.86 (23.66, 28.48)	0.016
Hypertension, n (%)	157 (63.3)	124 (59.3)	0.439
Shock, n (%)	27 (10.9)	20 (9.6)	0.759
Males, n (%)	175 (70.6)	172 (82.3)	0.005
WBC (10^9^/L), median (IQR)	12.75 (10.15, 15.78)	12.27 (9.52, 14.95)	0.087
Neutrophil(10^9^/L), median (IQR)	10.84 (8.66, 13.96)	10.43 (7.54, 13.16)	0.037
Lymphocyte(10^9^/L), median (IQR)	0.92 (0.65, 1.25)	1.04 (0.77, 1.44)	0.002
Monocyte (10^9^/L), median (IQR)	0.75 (0.48, 1.02)	0.69 (0.50, 0.95)	0.418
Hemoglobin (g/L), median (IQR)	139 (125, 149)	137 (127, 148)	0.777
Cr (μmol/L), median (IQR)	79.10 (62.08, 99.50)	74.20 (59.10, 96.50)	0.184
BUN, median (IQR)	7.16 (5.62, 8.47)	6.26 (5.14, 7.43)	<0.001
AST, median (IQR)	29.60 (23.62, 42.52)	27.40 (21.70, 37.90)	0.032
ALT, median (IQR)	29.60 (22.00, 42.42)	31.70 (22.80, 47.50)	0.321
Red cells transfusion (units)	4.75 (2.00, 8.62)	4.00 (1.50, 6.00)	<0.001
Plasma, median (IQR)	337.50 (0 600)	346.92 (0, 450)	0.297
Platelets (units), median (IQR)	10 (10, 20)	10 (10, 20)	0.819
Cryoprecipitate (units), median (IQR)	14 (9.20, 18)	10 (9, 18)	0.231
Bentall, n (%)	28 (11.3)	35 (16.7)	0.121
David, n (%)	13 (5.2)	7 (3.3)	0.450
CABG, n (%)	9 (3.6)	7 (3.3)	1.000
CPB duration, median (IQR)	201 (178.75, 240)	188 (164, 225)	0.002
ECMO, n (%)	9 (3.6)	7 (3.3)	1.000
Nadir Platelet, median (IQR)	50 (36, 62)	100 (87, 126)	<0.001
Stroke, n (%)	22 (8.9)	21 (10.0)	0.788
AKI requiring dialysis, n (%)	123 (49.6)	17 (8.1)	<0.001
Mortality during follow-up, n (%)	93 (37.5)	11 (5.3)	<0.001

## Results

### Baseline data

A total of 457 patients were finally enrolled. The last follow-up was done on April 14^th^, 2023. The median follow-up time was 16 [interquartile range 6-31] months. Compared to patients with postoperative thrombocytopenia (platelet nadir < 75 x 10^3^/µL), patients without postoperative thrombocytopenia were significantly younger and more often male (51 (43, 60) vs 56 (48, 64.25), *p* < 0.001. 82.3% vs 70.6%, *p* = 0.003, respectively). Their BMI was significantly higher (25.86 (23.66, 28.48) vs 25.03 (22.49, 27.68), *p* = 0.016). And after IPTW, these covariates were adjusted with no significant difference. They also had significantly less packed red blood cells transfusions (4.00 (1.50, 6.00) vs 4.75 (2.00, 8.62), *p* < 0.001). The frequency of AKI requiring dialysis and mortality differed significantly between groups. Patients with postoperative thrombocytopenia had higher rates of AKI requiring dialysis and mortality (Table 1). Multiple organ dysfunction syndrome (MODS) was the leading cause of the 104 deaths at the period of the study (48.1%; *n* = 50). Circulatory failure was the second major cause (21.2%, *n* = 22). Furthermore, septic shock mortality accounted for 7.7% of all deaths (*n* = 8), and unknown cause mortality consist of 8.7% (*n* = 9). Other cause-specific mortality was stroke, gastrointestinal hemorrhage, and rupture of aortic dissection aneurysm in 5.8% (*n* = 6), 6.7% (*n* = 7), and 1.9% (*n* = 2), respectively (Table 2).

**Table 2.** Perioperative data after inverse probability of treatment weighting.

	Group A*(*N* = 464)^†^	Group B (*N* = 472)^†^	*P* value
Age (year)	54.33 ± 12.27	54.17 ± 12.59	0.91
History of stroke, n (%)	24 (5.2)	19 (4)	0.56
Smoke, n (%)	98 (21.1)	95 (20.1)	0.81
DM, n (%)	12 (2.5)	11 (2.4)	0.90
COPD, n (%)	9 (1.8)	7 (1.6)	0.81
Intubation, n (%)	7 (1.5)	16 (3.5)	0.43
BMI	25.79 ± 4.11	25.52 ± 3.96	0.56
Hypertension, n (%)	286 (61.6)	271 (57.4)	0.45
Shock, n (%)	50 (10.8)	60 (12.7)	0.65
Males, n (%)	342 (75.8)	374 (77.3)	0.6
WBC (10^9^/L)	12.9 ± 4.44	13.52 ± 4.68	0.26
Neutrophil (10^9^/L)	10.95 ± 4.2	11.6 ± 4.5	0.23
Lymphocyte (10^9^/L)	1.12 ± 0.66	1.11 ± 0.62	0.86
Monocyte (10^9^/L)	0.78 ± 0.41	0.81 ± 0.41	0.57
Hemoglobin (g/L)	136.76 ± 18.52	136.4 ± 22	0.88
Cr (μmol/L)	84.19 ± 37.39	88.17 ± 40.96	0.4
BUN	7.05 ± 2.57	7.46 ± 4.03	0.5
AST	38.03 ± 31.8	45.92 ± 50.21	0.22
ALT	37.58 ± 27.63	44.75 ± 48.02	0.18
Red cells transfusion (units)	5.33 ± 4.07	4.52 ± 3.65	0.04
Plasma	350.43 ± 403.34	303.04 ± 348.94	0.24
Platelets (units)	13.99 ± 5.33	14.43 ± 5.45	0.46
Cryoprecipitate (units)	17.27 ± 57.09	13.16 ± 5.13	0.22
Bentall	52 (11.3)	72 (15.4)	0.23
David	26 (5.5)	20 (4.2)	0.59
CABG	19 (4)	13 (2.8)	0.51
CPB duration	212.94 ± 61.83	196.88 ± 48.61	<0.01
ECMO, n (%)	18 (3.8)	11 (2.3)	0.35
Nadir Platelet	49.93 ± 16.16	109.5 ± 36.23	<0.01
Stroke, n (%)	34 (7.4)	62 (13.1)	0.08
AKI requiring dialysis, n (%)	220 (47.3)	43 (9.2)	<0.01
Follow-up mortality, n (%)	175 (37.7)	22 (4.7)	<0.01

### Prognostic factors associated with morbidity and morbidity

The consequences of the multivariate analyses were presented in Table 3 (crude and after IPTW adjustment). In the crude model, the platelet nadir for postoperative stroke was significant (HR = 0.990, 95%CI (0.981-0.999), *p* = 0.031), but after IPTW adjustment, it was not significant (HR = 0.999, 95%CI (0.990-1.009), *p* = 0.889) (Table 3). Both the crude model and the IPTW modified model included platelet nadir as prediction indicators for AKI necessitating dialysis (HR = 0.980, 95%CI (0.975, 0.986), *p* < 0.001; HR = 0.979, 95%CI (0.971, 0.986), *p* < 0.001, respectively). Both in the IPTW adjusted model and the unadjusted model, it was the same as follow-up mortality.

**Table 3.** Relationship between platelet nadir and early-term mortality and morbidity in patients with TAAD derived from a generalized additive mixed model (GAMM).

Unadjusted			IPTW Adjusted*	
Stroke	HR (95%CI)	*P*-value	HR (95%CI)	*P*-value
Platelet Nadir^^^	0.990 (0.981, 0.999)	**0.031**	0.999 (0.990, 1.009)	0.889
AKI	HR (95%CI)	*P*-value	HR (95%CI)	*P*-value
Platelet Nadir^^^	0.980 (0.975, 0.986)	**<0.001**	0.979 (0.971, 0.986)	**<0.001**
Mortality	HR (95%CI)	*P*-value	HR (95%CI)	*P*-value
Platelet Nadir^^^	0.970 (0.963, 0.977)	**<0.001**	0.968 (0.960, 0.977)	**<0.001**

* The multivariate model adjusted for age, gender, stroke history, smoke, diabetes mellitus, body mass index, hypertension, shock, intubation entering the operative room, CPB duration, and platelet transfusion.

^^^ If mortality or morbidity preceded the nadir platelet count within 72 h postoperatively, we used the lowest platelet values ahead of those as predictors in multivariate analyses.

**Figure 1.**
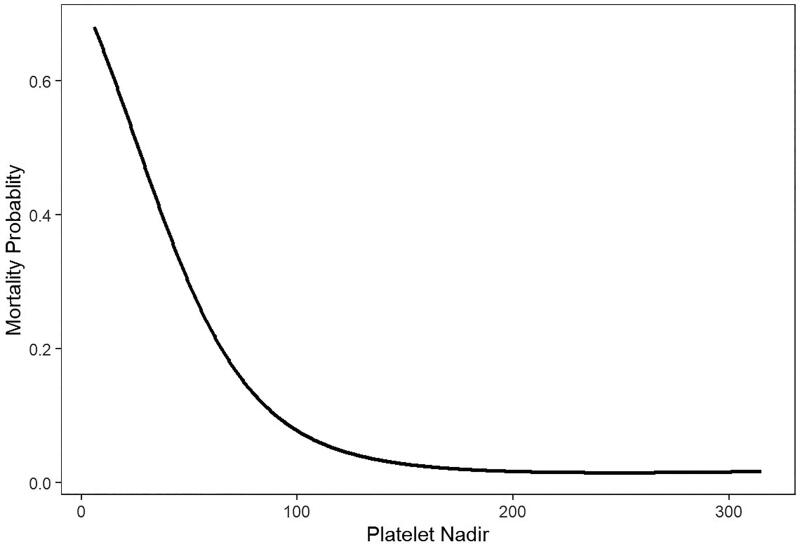
In a generalized additive mix model (GAMM) adjusted for age, gender, stroke history, smoke, diabetes mellitus, body mass index, hypertension, shock, and intubation entering the operating room, CPB time, a nonlinear relationship between platelet nadir and mortality probability during follow-up was detected.

It was the same as follow-up mortality both in the unadjusted and IPTW adjusted model (HR = 0.970, 95%CI (0.963, 0.977), *p* < 0.001; HR = 0.968, 95%CI (0.960, 0.977), *p* < 0.001, respectively). Figure 1 also showed that in GAMM adjusted for age, gender, stroke history, smoke, diabetes mellitus, body mass index, hypertension, shock, intubation entering the operating room, CPB duration, and platelet transfusion, a nonlinear relationship between platelet nadir and mortality probability was detected. As the value of platelet nadir increased, the probability of mortality decreased.

### Analyses that are stratified by important covariables

**Figure 2.**
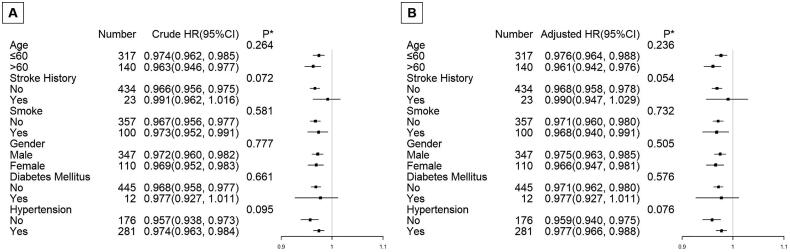
Subgroup analyses on effect modification of platelet nadir on follow-up survival in the crude model and adjusted model. *P value for interaction between two variables (platelet nadir and subgroup variables). The multivariate model adjusted for age, gender, stroke history, smoke, diabetes mellitus, body mass index, hypertension, shock, intubation entering the operative room, CPB duration, and platelet transfusion. None of the stratified variables, including age, gender, stroke history, smoke, diabetes mellitus, body mass index, hypertension significantly modified the effect of platelet nadir on follow-up survival; the p values of all interaction test were >0.05.

We conducted stratified analyses by subgroups defined by key covariables known to affect mortality, including age, gender, stroke history, smoking, hypertension, and diabetes mellitus in order to help increase power in testing for the overall effect of platelet nadir on follow-up mortality in the GAMM. Both the adjusted model and the crude model were used for all analyses. Figure 2 showed two patterns that were remarkably similar across subgroups: an increase in platelet nadir was associated with a considerable decline in follow-up mortality. Age, gender, stroke history, smoking, diabetes mellitus, or hypertension were all stratified variables, however, none of them significantly changed the impact of platelet nadir on survival (the p values of all interaction test were >0.05).

### IPTW-adjusted Kaplan–Meier analysis of overall survival

**Figure 3.**
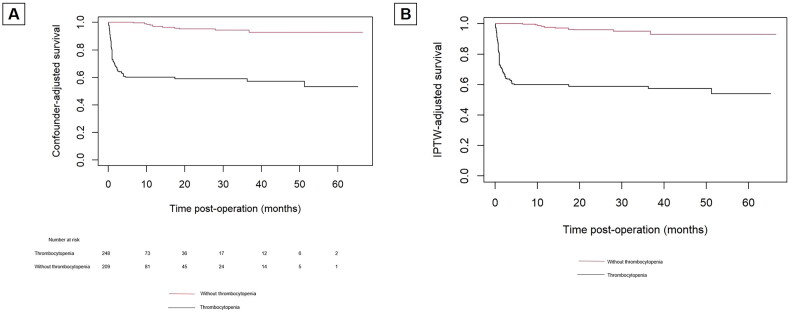
Confounder and IPTW-adjusted Kaplan–Meier survival curves in patients with thrombocytopenia and without after surgery for ATAAD.

Confounder-adjusted Kaplan-Meier curve showed patients without postoperative thrombocytopenia had a higher incidence of survival than those with postoperative thrombocytopenia (94.7% vs 62.5% *p* < 0.01). In IPTW-adjusted Cox proportional hazards analysis, postoperative thrombocytopenia not acquired was associated with a reduced risk of follow-up mortality (HR 0.067 95% CI: 0.036-0.125, *p* < 0.001), which was consistent with confounder-adjusted Kaplan-Meier curve (‘postoperative thrombocytopenia not acquired’ vs ‘postoperative thrombocytopenia’; HR: 0.086 [95% CI: 0.045-0.163]; *p* < 0.01) analysis (Figure 3).

### The ROC curve of postoperative platelet nadir for intermediate-term mortality

**Figure 4.**
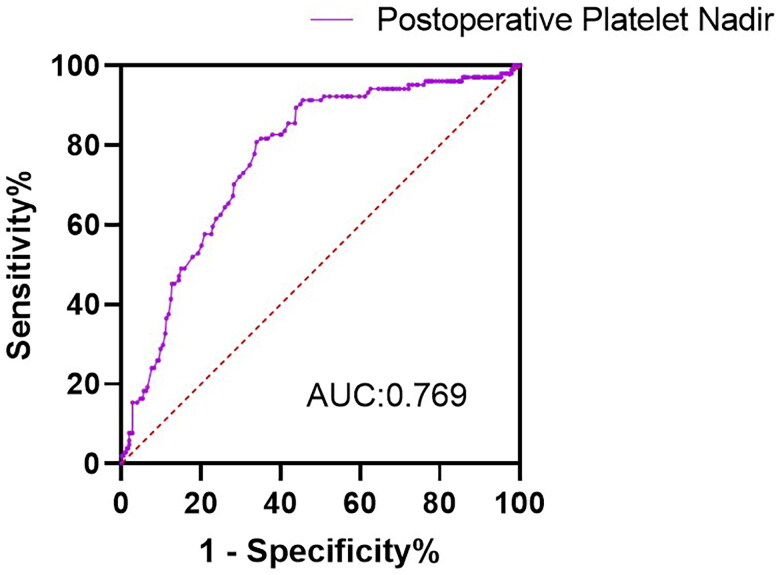
The ROC curve of postoperative platelet nadir for intermediate-term mortality.

The ROC curve for intermediate-term mortality of postoperative platelet nadir was shown in Figure 4. With optimal cutoff value of 65, postoperative platelet nadir exhibited sensitivity of 66%, specificity of 80.8%, and an AUROC of 0.769. The Hosmer-Lemeshow test revealed that the model’s fitness was acceptable (χ2 = 13.362; 8 degrees of freedom; *p* = 0.1).

## Discussion

The current investigation showed that postoperative AKI requiring dialysis and mortality were both significantly elevated by postoperative thrombocytopenia following open aortic surgery with median sternotomy, and a nonlinear relationship between platelet nadir and mortality probability was detected. Patients without postoperative thrombocytopenia had a better survival than those with postoperative thrombocytopenia. These findings lay the groundwork for additional studies looking into perioperative tactics intended to lessen postoperative thrombocytopenia, as well as a potential role for daily platelet monitoring following open aortic surgery with median sternotomy to identify patients with ATAAD at risk for negative outcomes.

Blood rushing through the tear at high pressure separates the layers after the intima of the aorta tears in ATAAD. The most important pathophysiological pathway, specifically the location and size of the initial intimal tear, which disrupts the media layer of the aortic wall, as well as the size of the affected aortic segments, determines whether a patient requires immediate surgical procedure, an interventional invasion, or perhaps the highest quality medical treatment [13]. The vascular walls segments separate as a consequence of the rip, and two or even more perfused channels may emerge within the aorta as a result of the bleeding that occurs both inside and along the aortic wall. The key cells in hemostasis are platelets. In certain physiological and pathological situations, platelets exhibit thrombo-inflammatory capabilities that link hemostatic and immunological responses by interacting with leukocytes, which can contribute to the pathogenesis of vascular disorders [14]. As for improvements in surgical techniques, individuals increasingly survive operations progressively and frequently. The common side effect of CPB surgery is postoperative thrombocytopenia. Its occurrence is greater than 30%, according to reports [15]. Platelet nadir often happens 48–72 h after surgery [15]. Based on that, platelet values were collected each day after surgery in each patient undergoing surgical repair of aortic dissection in the first postoperative three days.

During aortic operative treatment, patients with ATAAD typically develop impaired hemostatic capacity as a form of postoperative thrombocytopenia, which enhances the mortality rate associated with blood transfusion after cardiac surgery [16]. More and more, platelets are understood to have functions outside of thrombosis and hemostasis. Postoperative thrombocytopenia and disseminated intravascular coagulation (DIC) are frequent side effects of sepsis [17]. Because NETs on the surface of thrombus formation capture decreasing platelets from circulating blood, which in turn stimulated the development of thrombosis by interacting with neutrophils [18]. Thrombosis may result in tissue hypoperfusion, which, to some extent, results in inadequate tissue oxygenation and, ultimately, organ failure. In the present study, platelet nadir, modeled as a continuous variable, was inversely associated with postoperative AKI requiring dialysis, and follow-up mortality (HR = 0.979, 95%CI (0.971, 0.986), *p* < 0.001; HR = 0.968, 95%CI (0.960, 0.977), *p* < 0.001, respectively).

Stroke is commonly seen after open aortic repair of ATAAD [19]. In the present study, the incidence of stroke was 8.8%. Stroke is the second foremost cause of death worldwide. Platelets were contained in thrombus composition in acute ischemic stroke. The capital function of platelets is essential for hemostasis in the ischemic stroke [20]. Moreover, decreased platelets lead to an increased risk of bleeding, which in turn can lead to acute intracerebral hemorrhage. However, inconsistent with previous studies, platelet nadir as a continuous variable was not inversely associated with stroke following IPTW adjustment (HR 0.999 95% CI (0.990, 1.009), *p* = 0.889).

Another frequent surgical complication in ATAAD is AKI [21]. The prognosis of postoperative patients with ATAAD may be impacted by AKI, which could result in a substantially worse outcome than a single ATAAD. Surgery after CPB negatively affects the microcirculatory system [22], and platelet counts following surgery have recently been associated with reduced sublingual microcirculation [23]. As a result, AKI may result from reduced microvascular flow in the kidneys. In spite of this, a significant number of chronic hemodialysis cases showed that the platelet count decreased significantly (by 50% or more) during dialysis [24], but patients in the current study experienced acute kidney injury rather than chronic kidney disease, and the platelet nadir we measured before AKI occurred, which improved the reliability of the conclusion. In the present study, the incidence of AKI requiring dialysis was 30.6%. Different from previous studies, the AKI in this study was not according to the criteria of AKI defined in KIDIGO [25], but as described above, if a patient had an anomalous urine volume or blood creatinine levels after surgery and had AKI that required dialysis, we would consult a qualified nephrologist before deciding whether to perform dialysis.

## Limitations

The limitations of the present study are as follows: (1) This was a retrospective study, and it is inevitable for all inherent biases of retrospective analysis. Although we adjusted for some potential confounding factors, some unmeasured factors remained. (2) Additionally, data on medications that may contribute to postoperative thrombocytopenia administered to treat infection are lost.

## Conclusion

Early postoperative thrombocytopenia is an independent risk factor for morbidity and mortality in ATAAD. Since postoperative thrombocytopenia can herald a poor prognosis, perioperative strategies to prevent postoperative thrombocytopenia are strongly needed.

## Data Availability

Individual data used for this study are not publicly available for proprietary reasons.
